# RE: Promethazine is not a good option to aid sleep quality, especially for people using psychiatric services

**DOI:** 10.1192/bjb.2025.31

**Published:** 2025-06

**Authors:** Marta Corti, Aized Raza Shahbaz, Mai Elsawaf, Alice Roberts, Sophie Flood

**Affiliations:** 1 Psychiatry Core Trainee, NHS Greater Glasgow and Clyde, Glasgow, UK; 2 Psychiatry Core Trainee, NHS Forth Valley, Stirling, UK; 3 General Adult Psychiatry Registrar, NHS Greater Glasgow and Clyde, Glasgow, UK

## Use of promethazine from the perspective of resident psychiatry doctors

This paper provides valuable insights into the effects of promethazine and its increasing use to aid sleep, which we have seen in our own practice. As resident doctors in psychiatry who have in-patient duty shifts out of hours, we are frequently asked to prescribe medication for patients who can’t fall asleep. Promethazine is one of the commonly used medications. As noted in the paper, it is perceived to be ‘safer’ and less addictive than other options. It was useful to be reminded of the side-effects, as well as the need to check electrocardiograms before prescribing and to have a greater awareness of the impact promethazine may have on older patients.

We hope to offer our perspective on insomnia in acute in-patient treatment. Cognitive–behavioural therapy for insomnia (CBT-I), although noted to be highly effective,^1^ is not feasible in this setting. Even if it were available, patients are often too unwell or distressed to engage with it. A common scenario includes being called in the early hours of the morning by a nursing colleague to provide medication for a patient who is in despair owing to an inability to sleep. This is often a result of severe mental illness, such as psychosis, mania or agitation related to depression. In this scenario, when insomnia is linked to acute mental illness, promethazine has been a useful medication in our experience. There is also evidence to suggest that alternatives such as z-drugs are less effective in patients with a history of benzodiazepine misuse, as these patients develop tolerance.^2^

We note the distinction made between sedation and sleep quality. In the acute in-patient setting, is sedated sleep preferable to no sleep at all? This was acknowledged in the paper, which states that promethazine has value in the short term for people in acute psychiatric crisis. Moreover, research posits that the two phenomena of sedation and sleep are perhaps not as distinct as the author suggests.^3^

We agree that promethazine is not a long-term solution with respect to sleep quality. We found it interesting to read that promethazine has an addictive quality, that it has some street value and that it is used with codeine. Of note, there has been an increasing trend of antihistamine-related deaths.^4^ We need to keep this in mind when we discharge patients. As mentioned by the author, there can be withdrawal symptoms associated with cessation of promethazine and rebound insomnia. It is important to follow up and provide patients with psychoeducation on this.

When a patient is discharged and is more well, it is possible to consider long-term solutions such as CBT-I. Our health boards provide computerised CBT-I, although its success will depend on patient engagement. This can vary and will be affected by mental illness.

In conclusion, we agree with the author that there should be some caution with the use of promethazine. However, in the in-patient setting, it provides numerous benefits. As a result of reading this paper, we will aim to ensure promethazine is prescribed for a short course and be more proactive in offering CBT-I to our patients.
